# Prior and Present Evidence: How Prior Experience Interacts with Present Information in a Perceptual Decision Making Task

**DOI:** 10.1371/journal.pone.0037580

**Published:** 2012-05-29

**Authors:** Muhsin Karim, Justin A. Harris, John W. Morley, Michael Breakspear

**Affiliations:** 1 School of Psychiatry, Faculty of Medicine, University of New South Wales, Sydney, Australia; 2 The Black Dog Institute, Sydney, Australia; 3 School of Psychology, The University of Sydney, Sydney, Australia; 4 School of Medicine, University of Western Sydney, Sydney, Australia; 5 Queensland Institute of Medical Research, Brisbane, Australia; 6 Royal Brisbane and Women’s Hospital, Brisbane, Australia; McMaster University, Canada

## Abstract

**Background:**

Vibrotactile discrimination tasks have been used to examine decision making processes in the presence of perceptual uncertainty, induced by barely discernible frequency differences between paired stimuli or by the presence of embedded noise. One lesser known property of such tasks is that decisions made on a single trial may be biased by information from prior trials. An example is the time-order effect whereby the presentation order of paired stimuli may introduce differences in accuracy. Subjects perform better when the first stimulus lies between the second stimulus and the global mean of all stimuli on the judged dimension (“preferred” time-orders) compared to the alternative presentation order (“nonpreferred” time-orders). This has been conceptualised as a “drift” of the first stimulus representation towards the global mean of the stimulus-set (an internal standard). We describe the influence of prior information in relation to the more traditionally studied factors of interest in a classic discrimination task.

**Methodology:**

Sixty subjects performed a vibrotactile discrimination task with different levels of uncertainty parametrically induced by increasing task difficulty, aperiodic stimulus noise, and changing the task instructions whilst maintaining identical stimulus properties (the “context”).

**Principal Findings:**

The time-order effect had a greater influence on task performance than two of the explicit factors–task difficulty and noise–but not context. The influence of prior information increased with the distance of the first stimulus from the global mean, suggesting that the “drift” velocity of the first stimulus towards the global mean representation was greater for these trials.

**Conclusions/Significance:**

Awareness of the time-order effect and prior information in general is essential when studying perceptual decision making tasks. Implicit mechanisms may have a greater influence than the explicit factors under study. It also affords valuable insights into basic mechanisms of information accumulation, storage, sensory weighting, and processing in neural circuits.

## Introduction

Perceptual decision making tasks examine how subjects respond to a range of different stimuli in the presence of uncertainty. By manipulating the features of the stimuli or the nature of the task, it is possible to assess which effects most strongly influence behavioural outcomes of perceptual decision making processes. A number of different tasks across the visual, auditory and tactile modalities have been employed to this end. Vibrotactile discrimination tasks have been used in rodent [1], [2], monkey (for review see [3], [4]) and human subjects [5]–[9]. Participants are presented with a pair of vibrations typically in the flutter range (5–50 Hz) separated by an interstimulus interval (ISI). Subjects are asked to make an inference on the properties of the two stimuli, either by deciding which was faster, or by determining if the vibrations were the same or different. Subjects must thus make a comparison between the second vibration (Stim2) and their memory of the first vibration (Stim1) [10]. The percept-dependent decision is affected by a variety of stimulus properties – the frequency, amplitude and the resulting intensity [8], the temporal pattern of the stimuli [8], the duration of stimuli [11], and the duration of the ISI [5], [7], [12], [13].

Combined with imaging techniques including functional magnetic resonance imaging (fMRI) [5], [6], [9], [14], [15] and, in primates, single-cell electrophysiological recordings [16]–[22], three attributes of information processing are measured – the properties of the stimuli, the neural response, and the behavioural outcome. Explicit manipulation of either the physical properties of the sensory inputs or the task instructions allows elucidation of the most salient aspects of the sensory signals for perception, and how these vary with context [23]. Varying two or more factors together in a single factorial design offers the means to explore decision space, that is, the fundamental computational principles of how subjects make responses in discrimination tasks (for review see [24]).

Implicit influences of decision making also play an important role in such tasks and must be considered alongside explicit task factors. For instance, the “time-order effect” may exert a significant influence on perceptual decision making even if it is not an explicit factor in the task design. For a two-alternative forced choice (2AFC) task, accuracy and response time often systematically differ between the two possible presentation orders for each pair of stimuli, even when all other task factors are the same. Subjects tend to be more accurate when comparing a pair of stimuli if, on the dimension being judged (e.g. frequency), the first stimulus lies between the global mean of all stimuli and the second stimulus. Accuracy is worse if the first stimulus lies either above or below both the global mean and the second stimuli. These changes in accuracy based on the relative magnitudes and presentation order of stimuli are thought to arise from a “drift” in neural response towards the global mean, causing the two stimuli to be either perceptually further apart or closer together [5]. The relative importance of explicit factors versus implicit influences, and their putative interaction are poorly understood.

The objectives of this study are to quantify the relative influence of three explicit task factors on performance in comparison to the implicit time-order effect – that is, to characterise their relative strength and the presence of any putative interactions between these four factors. The three explicit factors studied were (1) Task difficulty (changing the frequency difference between pairs of vibrations), (2) Stimulus noise (degrading the temporal structure of vibrations), and (3) Task context, which was manipulated by requesting subjects to respond in counterbalanced sessions either to the question “Is the 2nd vibration faster?” (“fast-slow” context), or the question “Are the vibrations different?” (“same-diff” context). The former fast-slow question may be resolved via a simple magnitude subtraction between the estimated stimulus frequencies to compute the sign, whether positive or negative, upon which the decision is made. The latter same-diff question requires that subjects make a subtraction but also compare the magnitude of the subtraction to an internal standard of perceptual certainty for a “different” or “same” judgement and hence compute the precision of their perceptual beliefs.

Previous studies have measured the magnitude of the time-order effect directly for each subject [25], [26]. This permits a subject-wise comparison, and is of particular interest when combined with functional MRI data to examine how individual variations of the time-order effect vary with the BOLD response across different regions of the brain [5]. We instead quantified the influence of the time-order effect on task performance (accuracy and response time) across the explicit factors under investigation. We expected that the explicit factors under study–task difficulty, noise and context–would exert strong effects on both accuracy and response time, consistent with past observations [6], [8], [27]. However, the possible interactions between these factors have not been examined in the same vibrotactile discrimination task. Thus it is not clear whether the uncertainty induced by manipulation of one factor (such as an increased influence of perceptual noise induced by increased task difficulty) will influence the uncertainty associated with another factor (such as noise in the stimuli themselves). Nor is it known whether these sources of uncertainty will impinge on the implicit time-order effect. Independent effects between the factors would suggest that the brain may manage perceptual uncertainty across multiple cortical areas in parallel. Conversely, the presence of interactions would suggest cross-talk between, for example, the encoding of perceptual certainty and the perception of stimulus frequency. We studied the influence of these factors on behavioural correlates of decision making in a relatively large experimental cohort. After addressing this question, we then focus in detail on those factors potentially influencing the “drift” in the representation of stimuli that arises in the discrimination task.

## Results

### Experimental Overview

Sixty healthy human subjects completed a vibrotactile discrimination task over four sessions. The experiment was a partial 3 (task difficulty: easy, medium, hard) ×2 (noise: regular, noisy)×2 (context: fast-slow, same-diff) factorial design. Task context involved alternating the task instructions between a faster/slower and same/different command across separate sessions. The various factorial analyses reported below were confined to those arms of each factor that were fully populated. Prior to this main task, participants undertook an adaptive staircase procedure to titrate each participant’s ability to perform the task to average task difficulty target levels. By matching subject performance, the inter-subject variability was kept low on the main task factors whilst also avoiding floor and ceiling effects. This was achieved by deriving frequency difference values for each subject to target accuracy for easy trials at approximately 85% and hard trials at 65% proportion of correct responses for the fast-slow context (see Methods). Frequency differences for medium trials were chosen to be the geometric mean of those for the easy and hard trials. Pilot experiments demonstrated that accuracy for some of the possible trial-types (e.g. hard same-diff) were no greater than chance, thus 11 trial-types were presented (shown in Table 1).

**Table 1:** Trial-types of the vibrotactile discrimination task.

Context	Different/Same pairs	Easy		Medium		Hard	
Fast-Slow	Different	Regular	Noisy	Regular	Noisy	Regular	*NA*
		*24*	*24*	*24*	*24*	*24*	
	Same	*NA*	*NA*	*NA*	*NA*	*NA*	*NA*
							
Same-Diff	Different	Regular	Noisy	Regular	*NA*	*NA*	*NA*
		*24*	*24*	*24*			
	Same	Regular	Noisy	Regular	*NA*	*NA*	*NA*
		*24*	*24*	*24*			

The trial-types are determined by task difficulty (easy, medium, hard), noise (regular, noisy) and context (fast-slow, same-diff). There were 24 trials for each trial-type presented pseudorandomly across four sessions; two for the fast-slow context, and two for the same-diff context. Boxes with NA (not applicable) indicate possible trial-types that were not used in the study.

We present four distinct sets of analyses of these data. Analysis 1 focuses on the three explicit experimental factors; task difficulty, noise and context and their interactions, with a focus on how factors interact with increasing task difficulty. Performance across the three levels of task difficulty was examined, followed by task difficulty and noise (two levels each), and then task difficulty and context (two levels each). Analysis 2 addresses the influence of the time-order effect on subject accuracy as putatively expressed across the three levels of task difficulty (easy, medium, hard) in the fast-slow context. We confine this initial analysis of the time-order effect to this context as it is the most widely employed variant of vibrotactile decision making and because only this context contains all three levels of difficulty. Analysis 3 compares the relative influence on subjects’ performance of the three explicit factors and the implicit time-order effect, and examines potential interactions. This analysis is pooled across both contexts and is necessarily confined to the two levels of task difficulty (easy, medium) present in both contexts (see Table 1). Analysis 4 further addresses additional task factors influencing the time-order effect, focusing on how the distance between Stim1 and the global mean influences accuracy and speed of responses.

Subjects’ performance were assessed with two dependent variables – accuracy calculated as D-prime (d′), and the speed measure of response time (RT). For clarity of presentation, we present most statistics in table form, directing the reader to these in the text using curly brackets { }.

### Analysis 1: Task Difficulty, Noise and Context

Explicit task difficulty was manipulated by decreasing the frequency difference of the paired vibration stimuli. Confining our analysis to fast-slow trials confirmed that performance degraded as expected [12], monotonically with the three levels of increasing difficulty. Accuracy (d′) decreased (*F*_2,118_ = 41.083, *p*<0001) and response time slowed with increasing task difficulty (*F*_2,118_ = 15.390, *p*<0001).

For fast-slow, trial pairs with easy and medium frequency differences were also presented as noisy trials in which each stimulus was embedded within an aperiodic temporal structure (see Methods). Accuracy was significantly reduced for noisy vibrations compared to regular sinusoidal vibrations (*F*_1,59_ = 7.012, *p* = 0104), consistent with prior studies [8], [23]. Interestingly, there was no significant increase in response time for noisy trials (*F*_1,59_ = 2.798, *p* = 0997). There were no interactions between task difficulty and noise for d′ (*F*_1,59_ = 0.216, *p* = 6437) nor response time (*F*_1,59_ = 1.704, *p* = 1969).

**Figure 1:**
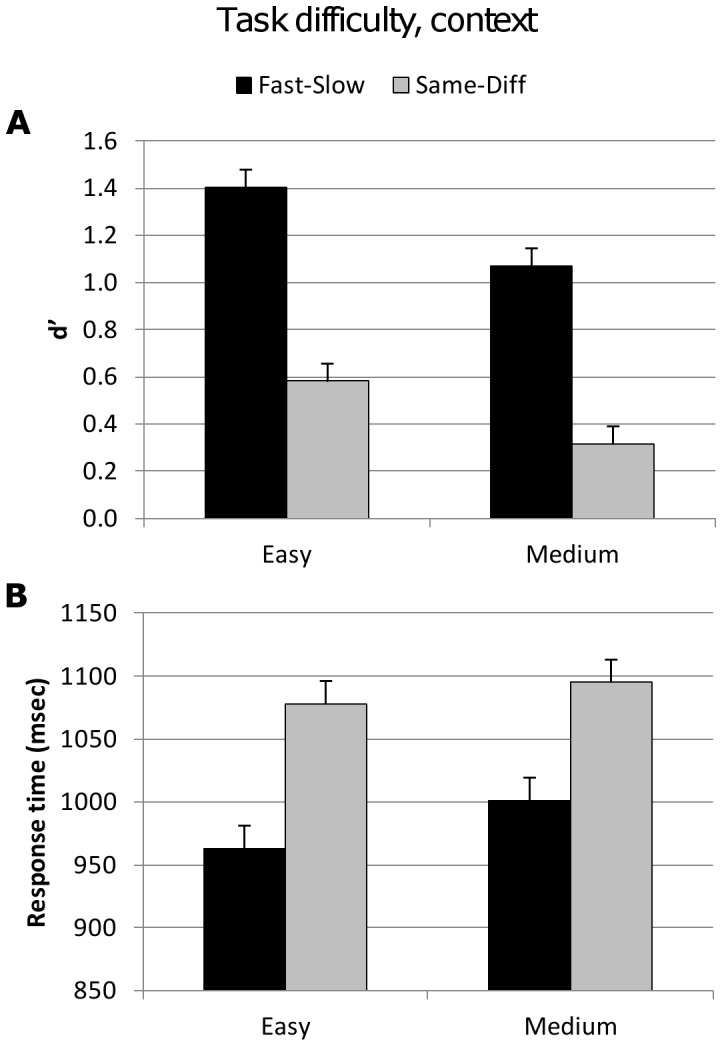
Accuracy (d′) and response time for explicit factors task difficulty and context. Vertical bars represent within-subject SEM. **A.** Top figure shows d′ values significantly decreased for the same-diff context trials compared to the fast-slow context trials. **B.** Lower figure shows significantly longer response times for same-diff trials.

The same-diff task required subjects to decide whether vibration pairs were the same or different. Both the fast-slow and the same-diff contexts are 2AFC tasks, allowing the classification of hits and false alarms based on participants’ choices to target/distracter trials. Thus, d′ was determined for the same-diff context in a similar fashion to that of the fast-slow context (see Methods, Procedure), and these accuracy values across contexts were analysed. Accuracy was significantly lower when subjects were required to decide if Stim2 was different from Stim1 (same-diff context) rather than deciding if Stim2 was faster than Stim1 (fast-slow context) (*F*_1,59_ = 225.149, *p*<0001, Figure 1a) with a corresponding increase in response time (*F*_1,59_ = 67.677, *p*<0001, Figure 1b). There was a significant sub-additive interaction between task difficulty and context for response time (*F*_1,59_ = 4.577, *p* = 0366) where subjects displayed a diminished difference in response time across easy and medium task difficulties for the same-diff context compared to the fast-slow context trials (Figure 1b).

In summary, performance across the three explicit task factors was affected in the expected directions, with lower accuracy with an increase in perceptual uncertainty for all three factors (task difficulty, noise and context). Responses are slower with increased task difficulty and with the same-diff context, but not for noise, despite the fall in accuracy. The absence of interactions between factors suggests that all of the sources of uncertainty act independently, except for task difficulty and context, which have an interactive (sub-additive) effect on the speed of response.

### Analysis 2: Evidence for the Time-Order Effect

**Figure 2:**
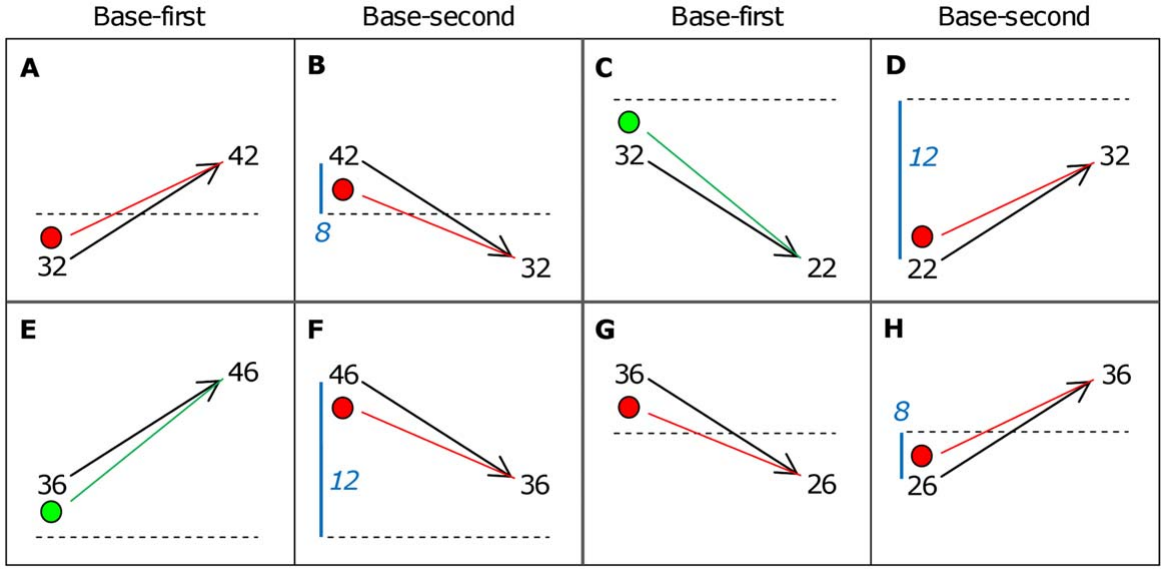
Classification of time-order trials based on the orientation of stimuli. The reversed presentation order of the same stimuli are in panel pairs: A with B, C with D, E with F and G with H. Each box shows the first vibration (Stim1) followed by an arrow indicting the second vibration (Stim2) to the right. The horizontal dashed line is the global mean of 34 Hz. Green dots (panels C and E) indicate where Stim1 “drifts” towards the global mean, and thus away from the Stim2 representation, classified as a preferred time-order trial. Red dots (panels A, B, D, F, G, H) indicate where Stim1 “drifts” towards both the global mean and the Stim2 representation, classified as a nonpreferred time-order trial. The vertical position of the red and green dots is used here to approximate the perceived frequency of Stim1 after it has drifted towards the global mean. Thus the slope of the dotted green and red lines reflects the difficulty in discriminating between Stim2 and the perceived Stim1. The distance between Stim1 and global mean are shown as blue numbered values referred to in Analysis 4 (panels B, D, F, H). In this subset of nonpreferred trials, some trials are “closer” to the global mean (i.e. 8 Hz) and other trials are “further” from the global mean (i.e. 12 Hz).

The potential influence of the time-order effect was first examined by comparing “preferred” to “nonpreferred” time-order trials in the fast-slow context. A likely interpretation of the time-order effect is that whilst the first stimulus is held in memory, its perceptual representation “drifts” towards the representation of the global mean (the average of the stimulus-set used in the task). Preferred trials are those where the representation of Stim1 drifts away from the representation of Stim2, causing the magnitude of the two vibrations to be perceived as more distinct. In contrast, nonpreferred trials occur when the Stim1 representation drifts towards Stim2, causing the vibrations to be perceived as less distinct [5] (see Figure 2).

Prior to the main task, participants underwent a titration procedure in which they were presented with pairs of vibrations until the adaptive staircases converged to average levels of accuracy performance matched across subjects. The average of the stimulus-set used was 34 Hz (see Methods, Analysis 2). Prior studies have noted that an internal standard needs as few as 15 to 20 trials to achieve a stable representation [5], [28]. Hence, a global mean of 34 Hz was likely established during the titration procedure and was present prior to subjects performing the main task. That is, the biasing of responses towards the global mean would likely have been present from the start of the main task. Figure 2 shows eight orientations that arise from two base frequencies (32 Hz and 36 Hz) used in the task with the position of the base and comparison stimuli being counterbalanced. The trials were accordingly classified as preferred or nonpreferred time-order trials. To compare the degree of influence each factor had on accuracy (d′), we use partial eta squared (η^2^) to estimate the variance explained by each factor (see Methods).

We found a strong and robust time-order effect in our data when down-sampling to the trials that permit a time-order analysis (that is, when restricting the analysis to the equal number of preferred to nonpreferred trials with matched frequency differences). Of note, the effect of time-order on d′ is quite profound amongst these trials, accounting for 51% of the accuracy (*F*_1,59_ = 60.533, *p*<0001, η^2^ = 0.51) compared to 22% for task difficulty across the three levels in this context (*F*_2,118_ = 16.924, *p*<0001, η^2^ = 0.22).

We also examined the data for any putative effect of the presentation order of the base frequency. This tests the possibility that the greater accuracy from the preferred trials may be due to the base frequency (32 and 36 Hz) being presented first (“base-first”) as opposed to second (“base-second”) for the nonpreferred trials (see Figure 2). To examine this, we down-sampled the cohort size to 44 subjects where sufficient numbers of counterbalanced "base-order" and "time-order" trials were present for all three task difficulty levels (see Figure S1 for details). These trials were all nonpreferred time-order trials which removed an influence of the time-order effect for the base-order comparison. Although the influence of explicit factor task difficulty remained strong within this smaller data-set, there was no trend towards an effect of the base-order (see Methods, Analysis 2).

### Analysis 3: The Time-Order Effect with the Three Explicit Factors

A formal, more-detailed comparison of the relative size of the time-order effect along with the task difficulty, noise and context factors was made where these factors were fully populated and counterbalanced. We report the accuracy and response time statistics of two analyses “Task difficulty, noise and time-order” and “Task difficulty, context and time-order” where each factor contributes two levels (task difficulty: easy and medium; noise: regular and noisy; context: fast-slow and same-diff).

#### Task difficulty, noise and time-order

**Figure 3:**
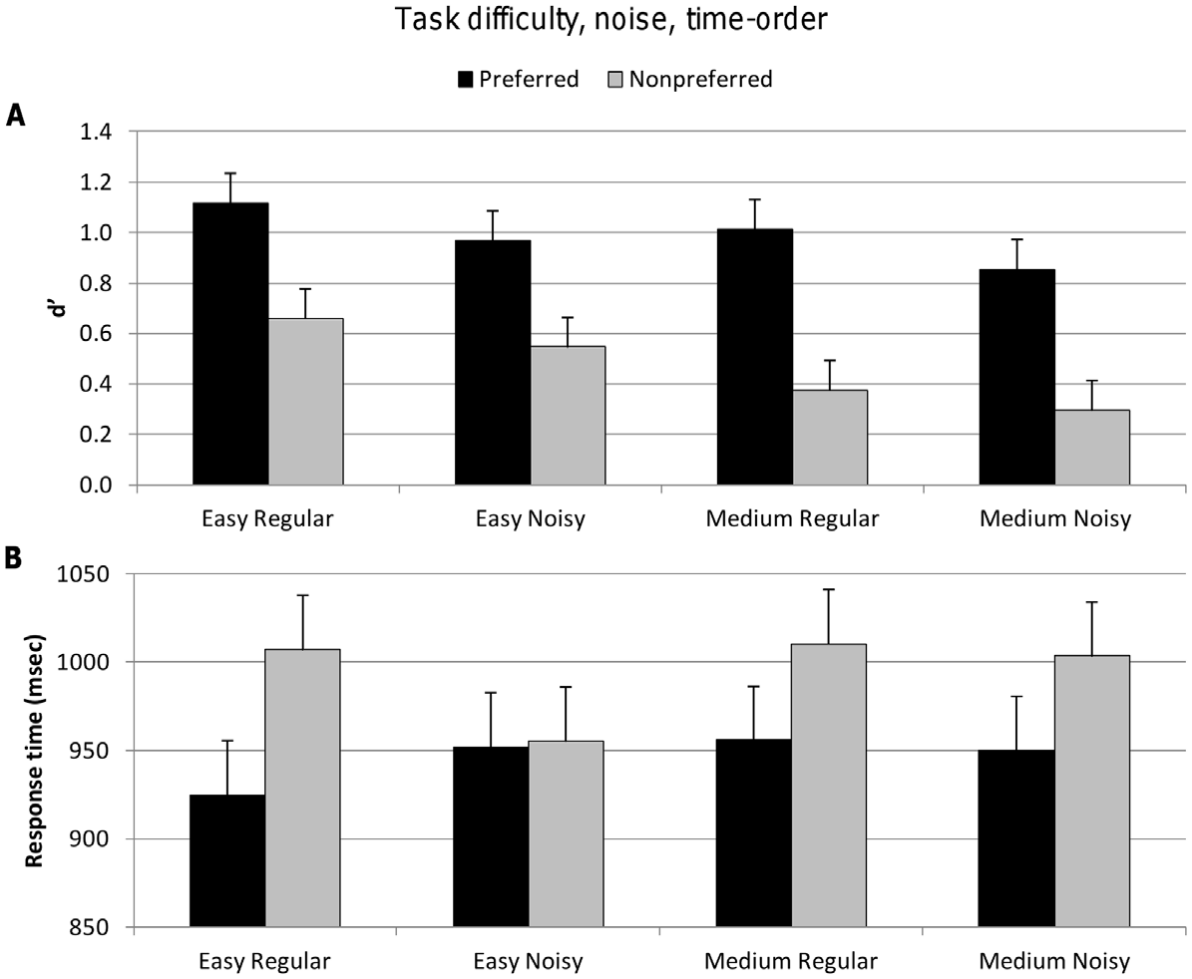
Accuracy (d′) and response time for explicit factors task difficulty, noise and implicit factor time-order. Vertical bars represent within-subject SEM. **A.** Top figure shows d′ values significantly decreased for the nonpreferred time-order trials, as it did for the explicit factors of task difficulty and noise. **B.** Lower figure shows significantly longer response times for nonpreferred time-order trials. There were no significant differences in response time across task difficulty and noise levels.

Analysis of these two explicit and one implicit factors in permissible trials revealed that there was once again diminished accuracy with increasing task difficulty^{2a}^ (easy versus medium), noise^{2b}^ (regular versus noisy) and time-order^{2c}^ (preferred versus nonpreferred). However, there was no significant interaction between these explicit and implicit factors on accuracy. That is, the presence of noise appeared to have no influence across the time-order trials (Table 2, Figure 3a).

**Table 2:** Statistics for task difficulty (easy, medium), noise (regular, noisy) and time-order (preferred, nonpreferred).

Factor	Dependent variable	F-statistic	p-value	Partial eta squared (η^2^)	Text reference
Task difficulty	d′	*F*_1,59_ = 15.114	*p* = .0003*	0.20	{2a}
Noise		*F*_1,59_ = 5.739	*p* = .0198*	0.09	{2b}
Time-order		*F*_1,59_ = 78.310	*p*<.0001*	0.57	{2c}
Task difficulty * Noise		*F*_1,59_ = 0.013	*p* = .9086	0	
Task difficulty * Time-order		*F*_1,59_ = 2.479	*p* = .1207	0.04	
Noise * Time-order		*F*_1,59_ = 0.271	*p* = .6044	0	
Task difficulty	RT	*F*_1,59_ = 3.292	*p* = .0747	0.05	{2f}
Noise		*F*_1,59_ = 0.528	*p* = .4704	0.01	{2d}
Time-order		*F*_1,59_ = 10.035	*p* = .0024*	0.15	{2e}
Task difficulty * Noise		*F*_1,59_ = 0.076	*p* = .7838	0	
Task difficulty * Time-order		*F*_1,59_ = 0.229	*p* = .6342	0	
Noise * Time-order		*F*_1,59_ = 2.753	*p* = .1024	0.04	

D-prime (d′) was used to assess accuracy and response time (RT) was used to assess speed.

There was no significant increase in the response time for noise^{2d}^, as reported above in the analysis ignoring time-order as a factor (Analysis 1). The effect of task difficulty on response time no longer reached statistical significance in this smaller set of trials^{2f}^, although subjects did spend significantly more time responding on nonpreferred trials than preferred time-order trials^{2e}^ (Figure 3b).

The time-order effect accounted for 57%^{2c}^ of the variance in d′ whereas task difficulty only accounted for 20%^{2a}^ and noise 9%^{2b}^. Thus, the time-order effect had a stronger influence on subject accuracy than task difficulty or noise. The time-order effect also had a greater influence on subjects’ response times than task difficulty or noise, with time-order accounting for 15%^{2e}^ of the variance (Table 2).

#### Task difficulty, context and time-order

Because same trials (where the Stim1 frequency is repeated) in the same-diff context cannot be classified as “preferred” or “nonpreferred” time-order trials, the three-way analysis between task difficulty, context and time-order must be confined to different trials only. Thus, we compared the fast-slow context trials (which by definition are all different) to the different trials of the same-diff context.

Strictly speaking, the time-order effect concerns the presentation order of the different stimuli (i.e. low-high or high-low) when task instructions refer explicitly to the order of the stimuli such as in the fast-slow context. For the same-diff context, participants do not respond to the order of the stimuli but rather assess whether the stimuli are the same or different (regardless of their order). Yet, the “drift” of the perceptual representation of the first stimulus might nonetheless influence performance. In particular, different trials could conceivably be perceived as more distinct (akin to preferred time-order) or less distinct (akin to nonpreferred time-order) depending on whether the first stimulus "drifts" toward or away from the second.

We tested for such an effect by undertaking a three-way analysis between task difficulty, context and time-order. An important caveat of this analysis concerns the assessment of performance and in particular, the estimation of d′. Because "same" trials in the same-diff context cannot be classified as “preferred” or “nonpreferred” time-order trials, this three-way analysis must be confined to different trials. However, false alarms in this context arise from mistakes on same trials, their removal precludes the use of d′, which is derived from hit rates and false alarm rates. Thus, we compared the fast-slow context trials (which by definition are all different) to the different trials of the same-diff context, and used proportion correct to assess accuracy. It is important to note that although the stimulus-sets for the two contexts are identical in this analysis, the exclusion of same trials from the same-diff context means that the full stimulus-set used by subjects to set their decision-criteria is not present. This issue, and the possible role of response bias in this analysis, are considered further in the Discussion.

**Figure 4:**
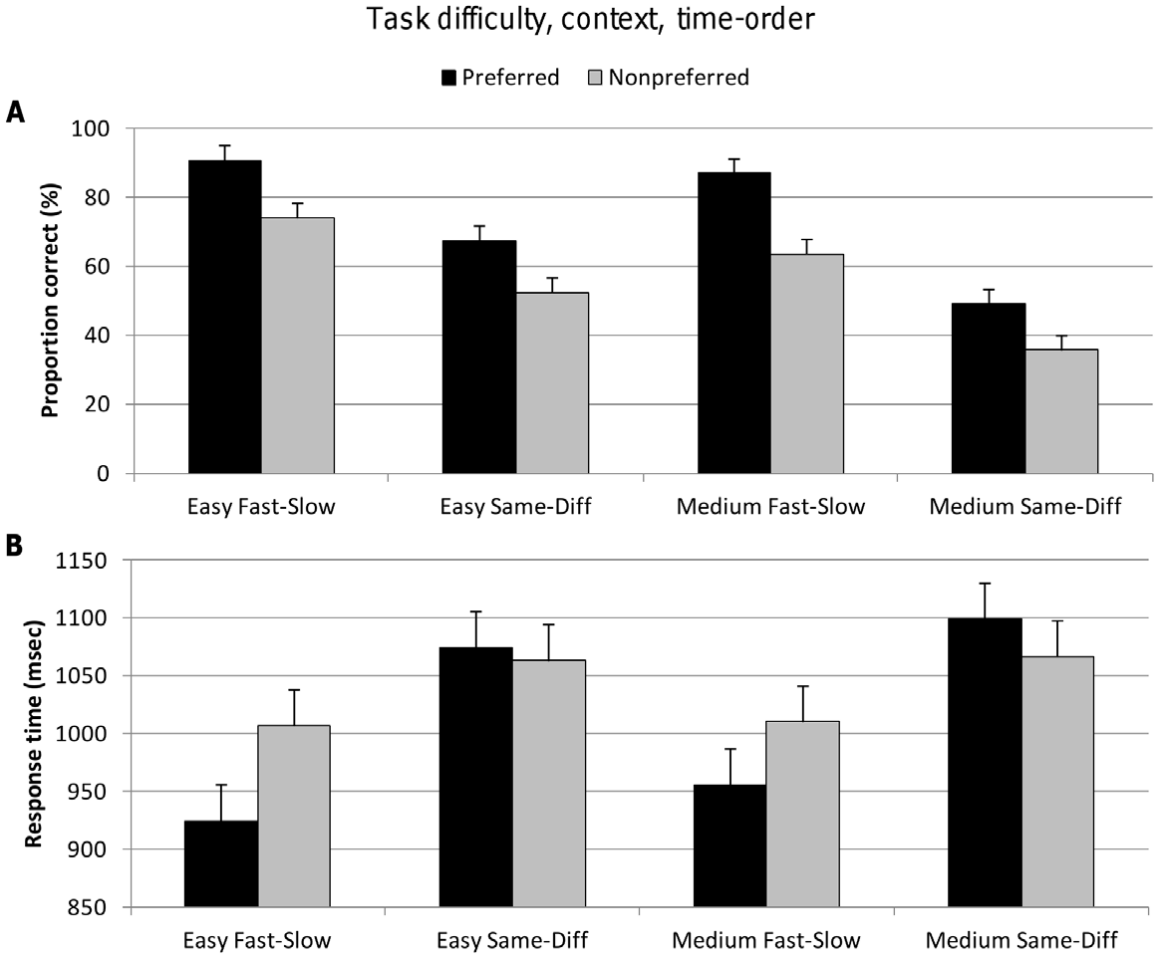
Accuracy (proportion correct) and response time for factors task difficulty, context and implicit factor time-order. Vertical bars represent within-subject SEM. **A.** Top figure shows a significant super-additive interaction between the task difficulty and context for proportion correct. **B.** Lower figure shows a significant increase in response time for the same-diff context compared to the fast-slow context trials. There was a significant interaction between context and time-order.

Even in this smaller subset of trials, there remained a significant effect on accuracy for the explicit factors of task difficulty^{3a}^ and context^{3b}^. The implicit factor of time-order exerted a strong effect on both contexts^{3c}^, hence confirming the proposition that perceptual “drift” operates on the different trials in the same-diff context, making trials either more or less distinct depending on their relationship to the global mean frequency (Table 3, Figure 4a). The explicit factor of context had a greater influence on accuracy than the implicit time-order factor^{3b, 3c}^. There was a robust interaction between context and time-order^{3d}^ for response time (Table 3). As evident in Figure 4b, the influence of the time-order effect on response times was confined to the fast-slow context, in contrast to its influence on accuracy which was present in both contexts.

**Table 3:** Statistics for task difficulty (easy, medium), context (fast-slow, same-diff) and time-order (preferred, nonpreferred).

Factor	Dependent variable	F-statistic	p-value	Partial eta squared (η^2^)	Text reference
Task difficulty	PC	*F*_1,59_ = 29.899	*p*<.0001*	0.34	{3a}
Context		*F*_1,59_ = 131.095	*p*<.0001*	0.69	{3b}
Time-order		*F*_1,59_ = 67.861	*p*<.0001*	0.53	{3c}
					
Task difficulty * Time-order		*F*_1,59_ = 0.412	*p* = .5234	0.01	
Context * Time-order		*F*_1,59_ = 2.235	*p* = .1402	0.04	{3e}
Task difficulty	RT	*F*_1,59_ = 1.522	*p* = .2223	0.03	
Context		*F*_1,59_ = 42.950	*p*<.0001*	0.42	
Time-order		*F*_1,59_ = 2.256	*p* = .1384	0.04	
Task difficulty * Context		*F*_1,59_ = 0.015	*p* = .9025	0	
Task difficulty * Time-order		*F*_1,59_ = 1.765	*p* = .1892	0.03	
Context * Time-order		*F*_1,59_ = 11.490	*p* = .0013*	0.16	{3d}

Proportion correct (PC) was used to assess accuracy and response time (RT) was used to assess speed. Note that in these contrasts, the same trials that make up half of the same-diff context has been excluded from analysis.

### Analysis 4: Distance between the First Stimulus and Global Mean

The previous analyses demonstrate that the implicit time-order effect has a strong influence on subject performance in this vibrotactile discrimination task. Further analysis of a subset of trials permits an additional examination –to investigate whether the magnitude of distance from Stim1 to the global mean influences performance. This in turn allowed the examination of whether the putative “drift” underlying the time-order effect has constant speed or whether it is dependent on the position of Stim1 in relationship to the overall stimulus-set.

Consider the panels B, D, F and H in Figure 2 where the frequency difference is 10 Hz between each pair of stimuli. For panels B and H, the distance (difference) between the global mean (34 Hz) and Stim1 is 8 Hz, whereas the corresponding distance for panels D and F is 12 Hz. Thus Stim1 is closer to the global mean in panels B and H, than for D and F. Hence each task difficulty level gives rise to one “closer” and one “further” trial-type, allowing a within-subject analysis of the difference in distance on task performance. This analysis was possible using the data from 44 out of the total of 60 subjects across the three levels of task difficulty (see Figure S1 for details). This distance comparison was restricted to nonpreferred time-order trials only, removing an influence of the time-order effect. A similar distance comparison amongst preferred trials was not possible in this study since the distance to the global mean was a fixed 2 Hz for all subjects (see Figure 2, panels C and E).

**Figure 5:**
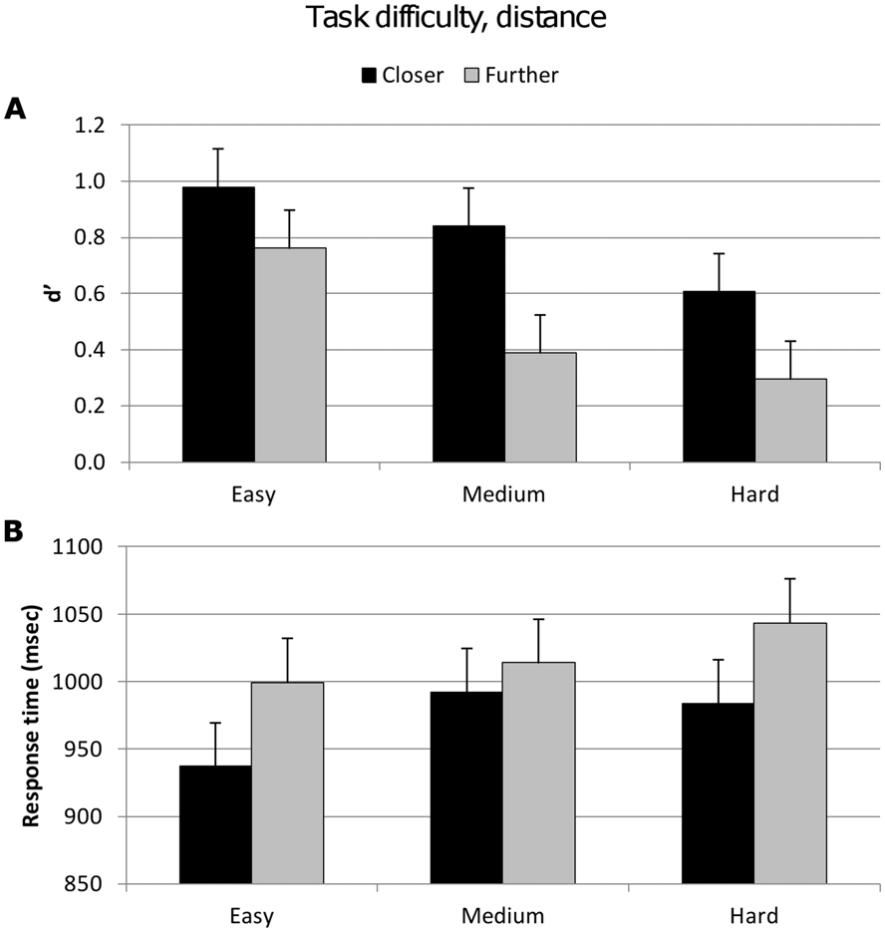
Accuracy (d′) and response time for factor task difficulty and implicit factor distance. Vertical bars represent within-subject SEM. **A.** Top figure shows d′ values significantly decreased with increasing task difficulty and for the further distance trials. **B.** Lower figure shows significantly longer response times for the further distance trials.

Table 4 shows there was a significant difference in accuracy between the closer and further distance trials^{4a}^. Subjects were more accurate for trials where the Stim1 and global mean were closer compared to trials where Stim1 and the global mean were further in distance (Figure 5a). The partial eta squared values show that the variance in response is almost equally accounted for by both task difficulty and distance^{4a, 4b}^ (25% and 29%, respectively). There was also a significant effect of this distance on response time^{4c}^. Subjects took a shorter time to respond for the closer distance trials compared to the further distance trials (Figure 5b). This distance effect explained substantially more of the response time variance than the explicit factor task difficulty.

**Table 4:** Statistics for task difficulty (easy, medium, hard) and distance (closer, further).

Factor	Dependent variable	F-statistic	p-value	Partial eta squared (η^2^)	Text reference
Task difficulty	d′	*F*_2,86_ = 14.155	*p*<.0001*	0.25	{4b}
Distance		*F*_1,43_ = 17.410	*p* = .0002*	0.29	{4a}
Task difficulty * Distance		*F*_2,86_ = 0.960	*p* = .3870	0.02	
Task difficulty	RT	*F*_2,86_ = 2.583	*p* = .0814	0.06	
Distance		*F*_1,43_ = 6.663	*p* = .0133*	0.13	{4c}
Task difficulty * Distance		*F*_2,86_ = 0.687	*p* = .5057	0.02	

D-prime (d′) was used to assess accuracy and response time (RT) was used to assess speed.

**Figure 6:**
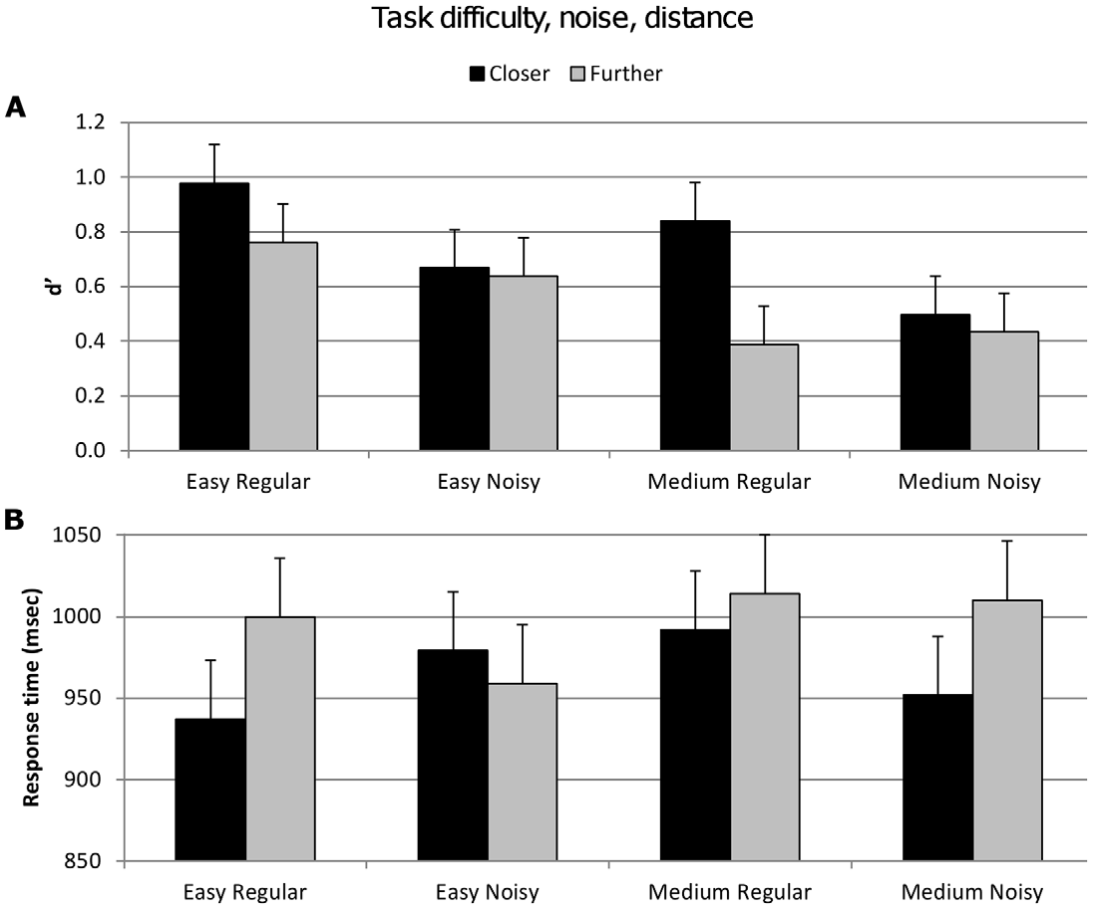
Accuracy (d′) and response time for factors task difficulty, noise and implicit factor distance. Vertical bars represent within-subject SEM. **A.** Top figure shows d′ values significantly decreased for with increasing task difficulty, presence of noise, and for the further distance trials. There was an interaction between noise and distance. **B.** Lower figure shows there were no significant main effects for response time.

A similar analysis with the same subset of participants (44 out of the total 60) was conducted on trials that contained aperiodic noise, allowing a three-way analysis of task difficulty (easy, medium), noise (regular, noisy) and distance (closer, further). This additional analysis showed that there was a significant interaction for accuracy between noise and distance^{5a}^. Subjects’ performance diminished more greatly between closer and further trials for regular vibrations compared to noisy vibrations (Table 5, Figure 6a). There was no significant difference in response time for noise^{5b}^ (Figure 6b).

**Table 5:** Statistics for task difficulty (easy, medium), noise (regular, noisy) and distance (closer, further).

Factor	Dependent variable	F-statistic	p-value	Partial eta squared (η^2^)	Text reference
Task difficulty	d′	*F*_1,43_ = 14.621	*p* = .0005*	0.25	
Noise		*F*_1,43_ = 8.031	*p* = .0070*	0.16	
Distance		*F*_1,43_ = 7.323	*p* = .0097*	0.15	
Task difficulty * Noise		*F*_1,43_ = 0.577	*p* = .4516	0.01	
Task difficulty * Distance		*F*_1,43_ = 1.119	*p* = .2961	0.03	
Noise * Distance		*F*_1,43_ = 5.577	*p* = .0228*	0.11	{5a}
Task difficulty	RT	*F*_1,43_ = 3.252	*p* = .0784	0.07	
Noise		*F*_1,43_ = 0.296	*p* = .5894	0.01	{5b}
Distance		*F*_1,43_ = 2.889	*p* = .0964	0.06	
Task difficulty * Noise		*F*_1,43_ = 0.607	*p* = .4403	0.01	
Task difficulty * Distance		*F*_1,43_ = 0.471	*p* = .4963	0.01	
Noise * Distance		*F*_1,43_ = 0.953	*p* = .3343	0.02	

D-prime (d′) was used to assess accuracy and response time (RT) was used to assess speed.

## Discussion

Vibrotactile discrimination tasks are ideally suited to examine decision making in the presence of different sources of uncertainty. Here we used three factors to induce uncertainty: (1) Task difficulty, (2) Noise, and (3) Context (where the task instruction is changed). All three factors exerted a significant influence in performance (affecting accuracy and reaction time). Context was the factor with most profound influence – performance decreased substantially when changing from a faster/slower to a same/different task. The fast-slow task can be achieved by a simple subtraction of the two inferred stimulus frequencies and a (forced) decision based on the sign of this difference. In contrast, the same-diff task requires encoding of both the likely stimulus frequency *and* the confidence of these estimates. The decision then requires at least two steps. First, estimating the difference of the frequencies normalised to their relative confidence (akin to a t-test). Second, judgement of whether this quantity exceeds an internal decision threshold. Hence the encoding and decision making are both computationally more complex in the same-diff context, presumably explaining the increased reaction time and diminished accuracy.

To our surprise, the explicit task factors were largely independent. The only interaction of note was the sub-additive effect of task difficulty and context on reaction time. The presence of largely independent effects could suggest that the human brain may disambiguate perceptual uncertainty across multiple cortical areas in parallel, at least across the range of task factors we employed. The sub-additive interaction between context and task difficulty on response speed is, however, of interest (Results, Analysis 1 and Figure 1b). Assuming subjects are performing close to Bayesian optimality [29], the large increase in response time across easy and medium trials in the fast-slow context suggests that the further accumulation of sensory evidence is crucial in maintaining reasonable accuracy when the stimuli are less distinct. The smaller increase in response time in same-diff trials as the stimuli become less distinct suggests that such a strategy is less optimal when attempting to detect change. One possibility is that the accumulation of evidence during presentation of the second stimulus is offset by loss of confidence in the first [30]. Whatever the underlying explanation, this interaction suggests that the cortical regions computing these two task factors do interact when “deciding when to decide” [31].

In addition to these three factors of interest, we demonstrated that the implicit time-order effect was having a profound influence on decision making in our task. First observed by Fechner in 1860 using a weight discrimination task, the time-order effect is a well-established phenomenon in psychophysics (for a review see [32]). Its influence has been implicated in a variety of magnitude discrimination tasks including weight-lifting judgements [33], [34], visual contrast discrimination [35], auditory loudness discrimination [36]–[39] and in stimulus duration discrimination tasks [37], [40]–[42]. Two studies have examined the time-order effect in vibrotactile discrimination tasks [5], [26]. In our study, the time-order effect exerted a stronger effect than either task difficulty or noise (but not context). Whilst the relative influence is naturally specific to our particular experimental manipulations (for example, we could have added more noise to noisy vibration pairs), we believe this finding is still of interest for a number of reasons, not the least of which is because the range of difficulty in our task varied from high levels of accuracy to near chance. Combining any two of our task factors (for example hard trials with noise) reduced performance to the level of chance. This range is at least as broad as many reported in the literature, including notable electrophysiological and functional neuroimaging studies where the time-order effect was not included as a task factor.

The implicit time-order effect appeared to be a largely independent factor. The one exception was the interaction between time-order and context, again a sub-additive influence on reaction time. Whereas response time increased for nonpreferred trials in the fast-slow context, there was no such increase in the same-diff context (Figure 4b). As in the case for the sub-additive interaction between task difficulty and context, there may be cortical cross-talk when finding the optimal time to decide. However, the neural networks responsible for combining current and prior information to bias subsequent decisions may do so independently from those neural regions that process changes in frequency difference or temporal patterns of vibrations.

The analysis of the time order effect in the same-diff context is of interest because the task instructions do not explicitly involve the temporal order of the stimuli. Indeed, it appears that the same underlying process of “perceptual drift” operates in the different trials of this context, effectively making some trials more distinct and others less so, depending on their relationship to the global mean frequency. Importantly, as noted in the Results, this analysis necessarily depends on assessing accuracy through proportion of correct responses, a dependent measure that is prone to response bias whenever subjects resort more often to one of the two responses if they are uncertain (and given that the same-diff comparisons are more difficult, response bias might exert a stronger effect here). However, even if a response bias was operating, it would have an equal influence on preferred and nonpreferred trials unless there was indeed a time-order effect. That is, a response bias alone cannot account for the significantly decreased accuracy for nonpreferred trials that we observed in this context, although it could in theory magnify the effect. However, the absence of a significant interaction between the time-order effect and context^{3e}^ suggests that the size of any putative response bias may have been limited.

We also observed a significant difference in accuracy and response time between closer and further trials even when both have the same frequency difference. What could account for this? In any given trial, the perceptual representation of the first stimulus is said to “drift” towards the global mean during the working memory maintenance interval [5]. This gives rise to preferred and nonpreferred time-order trials, as outlined in this study. The "diffusion model" (DM) has been developed in order to describe a number of behavioural observations from perceptual decision making studies [43]. The DM assumes a stochastic accumulation of sensory evidence over time, from a starting point to one of two decision boundaries corresponding to the two choices the subject is required to make in 2AFC tasks. The model decomposes accuracy and response times into components of processing that reflect the rate of evidence accumulation and the amount of evidence required to make a decision (starting point and decision boundaries) amongst other parameters (see [44]). Whilst the DM is used to describe the dynamic process of how subjects reach a decision, the model can also accommodate the so-called “drift” process of Stim1 representation towards the global mean during the ISI period. The lower accuracy for the “further” distance compared to the “closer” distance nonpreferred trials suggests that the drift rate of Stim1 representation was faster for these trials. This is consistent with classic accounts of drift and diffusion in statistical mechanics which model many phenomena as diffusion in a parabolic well [45]. Accordingly, the estimated frequency of Stim1 would drift down the side of a quadratic-shaped well during the maintenance period toward the global mean frequency. The further away from the mean, the steeper the slope and the faster the drift. The interaction between this distance effect and noise is also illuminating here as drift and diffusion rates are generally interdependent in these models. To explain the time-order effect, it would be necessary for an internal representation of this dynamic landscape to form whilst subjects learnt the global properties of the stimulus-set. However, since the ISI was fixed for each trial, we were not free to investigate the nature of “drift” with additional manipulations.

Whilst the notion of a drifting memory trace is heuristically appealing, there is no direct evidence for such an effect. It is, moreover, important to consider other plausible accounts for the time-order effect. We suggest a very simple alternative mechanism of sensory weighting that also provides a sufficient explanation of the performance results observed between the distance trials (and comparisons between preferred and nonpreferred time-orders). It is based on averaging the Stim1 magnitude and global mean magnitude to form the perceptual representation of Stim1 held in memory, which we will call the “perceived Stim1”. For example, for closer vibration pairs 42–32 and 26–36 (Figure 2, panels B and H), the perceived Stim1 are 38 and 30 Hz, respectively. The “perceived difference” that the subject uses to make their decision is the absolute value of the perceived Stim1 minus Stim2 which equals 6 Hz for both closer trials. In contrast, for further vibration pairs 46–36 and 22–32 (Figure 2, panels D and F), the perceived Stim1 are 40 and 28 Hz, respectively. The perceived difference for these further trials is 4 Hz. Hence, the perceived difference is greater for closer trials (i.e. 6 Hz) than the further trials (i.e. 4 Hz). Use of this sensory weighting approach would cause the stimulus pairs of closer trials to be perceived as more different, leading to more accurate and faster responses compared to further trials – consistent with the performance as displayed by our study participants.

According to this “sensory weighting approach”, there is no drift in the memory of the stimulus, but rather a weighting of that memory and the “best guess” (the global stimulus average) as a means of compensating for any loss of memory certainty. Different weights to source evidence from Stim1 and the global mean may occur. If these were instead weighted 60 to 40 (Stim1 to the global mean), then the closer trials would have a perceived difference of 6.8 Hz compared to the further trials of 5.2 Hz. Decisions where subjects experience longer elapsed times before making a response may allow the subject to weight prior information more than sensory evidence. This is the case when prior probability of reward is incorporated into the decision process as a dynamic bias signal that increases as a function of decision time [30].

This approach is derived from Hellström’s sensation weighting model [36], [37], [40], and incorporates work conducted by Michels and Helson [46]. In 2AFC tasks, decisions are made by comparing the perceptual representation of Stim2 to the memory/percept of Stim1, the latter of which may be a compromise between the Stim1 magnitude and the mean of the stimulus-set used in the task (see [47], [48]). Preuschhof et al. (2009), inferred that subjects formed an average representation and compared the second stimulus to a combination of this implicit average representation and the vibration frequency of the first stimulus [5], consistent with our study. This framework of perception based on sensation and prior experience can be traced back to von Helmholtz’s Treatise on Physiological Optics (1925) who noted that “previous experiences act in conjunction with present sensations to produce a perceptual image” [49]. Our results reiterate that perception in a simple discrimination task is greatly affected by prior experience.

There are a few study limitations that are important to consider. Firstly, for each subject, there were only four preferred trials and four nonpreferred trials used for comparison in performance (see Methods). Only this small subset of trials permitted this matched-magnitude comparison. However, we had a large number of subjects in this study where all 60 participants were used in most analyses (44 subjects were used when down-sampling was necessary). Due to the partial factorial structure of our experiment – imposed by time and task constraints – we cannot report all the results in a single analysis, but require down-sampling for some of the analyses in order to fully populate all arms of the corresponding factors. We hence report a number of separate, although not independent statistical analyses, introducing the potential for type I error. Many of our results are particularly strong and would easily survive conservative correction. Finally, the potential role of response bias – a proposed source of the time-order effect – was minimised in our study by appropriate counterbalance of trial-types and the employment wherever possible of d′ (and not proportion correct) as our principle measure of accuracy. We have noted above the potential role of response bias in the single analysis where proportion of correct responses was required as the dependent measure.

### Conclusion

Our data demonstrate that prior information has a strong influence on perceptual decision making as shown by our 2AFC task. Hellström has examined the nature of the time-order effect since the late 1970s and had stated that the influence of the time-order effect was largely being ignored [40]. It may not be possible to characterise the time-order influence in discrimination tasks as we have achieved in our study, which depends on experimental design, magnitude of base and comparison frequencies and how often the stimuli are presented. However, knowledge of how the time-order effect may influence decision making in a vibrotactile discrimination task, and perhaps other 2AFC tasks, is essential when designing an experiment. As we have shown, the time-order effect can exert a strong influence on behaviour, and without being properly modelled, would likely be a source of unaccounted variance in the data leading to poorer study power. Without proper counter-balancing, it could act as a strong confound. Finally, the time-order effect and the influential role of prior information in general is clearly a process of significant interest in its own right.

## Methods

### Materials

The vibrotactile stimulator (Dancer Design, St. Helens, UK) used piezoelectric bender elements to deliver the mechanical stimulus. Mechanical contact to the skin was made via a flat plastic tip 8 mm in diameter which was mechanically coupled to but electrically insulated from the bender element. A static surround with a hole 10 mm in diameter limited the stimulation to a region just under the contactor. All stimuli took the form of sinusoidal displacement waveforms. The vibrotactile discrimination task protocol was written in Matlab (version 2007b, Mathworks), using the Psychophysics Toolbox extensions [50], [51] and a National Instruments card (USB-6259, National Instruments) to drive the equipment. The stimulators were capable of delivering frequencies from 1 to 500 Hz with amplitudes up to 1 mm peak-to-peak (below 200 Hz) when supplied with a 10 V peak-to-peak input signal.

### Procedure

Participants gave written informed consent and the study was approved by the University of New South Wales Human Research Ethics Committee. Subjects were paid for their participation in the study. Sixty participants performed the task. The average age was 27.9 years (range: 19–61, standard deviation: 9.3). Thirty-four were male. All participants were right-handed. Self-reporting indicated that none of the subjects had a psychiatric disorder, neurological disorder, or any drug or alcohol dependence.

Participants first performed a titration to match performance across subjects. This was followed by the main task where subjects were presented with 11 trial-types (see Table 1). Subjects’ response to uncertainty was assessed with two dependent variables. For accuracy, D-prime (d′) was used. d′ is a measure of sensitivity that takes subjects’ response bias into account and is calculated as follows:
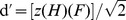
where (*H)* is the hit rate and (*F*) is the false alarm rate, both z-transformed. Trials where the second vibration was faster than the first were designated as target trials. A correct response to target trials was a hit, whereas an incorrect response in the absence of the target (distracter trials) was a false alarm. The hit rate was determined by dividing the number of hits by the number of trials where the target was present (hits and misses). The false alarm rate was determined by dividing the number of false alarms by the number of trials where distracters were present (false alarms and correct rejections). As this was a 2AFC task design (as opposed to a one-interval yes-no design), d′ values were adjusted downward by a factor of **√**2. Furthermore, adjusted d′ was used to account for cases of perfect accuracy which would otherwise result in an infinite d′. This was facilitated by adding 0.5 to all of the data cells (hits, misses, false alarms and correct rejections) [27]. For the same-diff context trials where subjects answered the question “Are the vibrations different?” when faced with different and same vibration pairs, d′ was determined in a similar fashion. For this context, trials where the vibration pairs were different were designated as target trials. A correct response to a target trial (correctly stating that vibrations were different) was a hit, whereas an incorrect response to distracter trials (same trials) was a false alarm.

The tactile stimulus was 512 milliseconds (msec) in duration with approximate amplitude of 280 µm peak-to-peak. On each given trial, the subjects compared two consecutive vibrations, separated by an interstimulus interval (ISI) of 600 msec. One of the vibrations was a set base frequency (32, 34 or 36 Hz), the other a comparison frequency. The subject’s right index finger pad was placed on the vibrotactile probe. The subject’s left middle and index fingers were placed on a keyboard left and right arrow response keys, respectively. Subjects were prompted to answer the context question in a Yes/No fashion. The left/right position of Yes and No was counterbalanced across subjects. Subjects had two seconds to respond as soon as the second vibration (Stim2) played and the response screen appeared. Subjects were instructed to answer quickly but as accurately as possible. The time in msec from the start of Stim2 until the subject made their response within the two second response period was logged as the response time for a trial. An incorrect response was logged if the response period lapsed without a key press from the participant. White noise was delivered from headphones to mask auditory cues during the experiments. Analyses were conducted using PASW 18.0 Statistical Package (SPSS, Inc., Chicago, Illinois). Repeated measures analyses of variance (ANOVA) was used to compare within-subject differences in behavioural performance across the different trial-types of the main task.

#### Titration procedure

Each subject’s frequency sensitivity was measured using an adaptive staircase procedure which automatically tailors the task difficulty to individual performance. The staircase used was a variation of the up-down transformed rule method [52]. The subjects had to answer the question, “Is the 2nd vibration faster?” by indicating Yes or No on the keyboard. Each trial contained one vibration, at the base frequency of 34 Hz, and a comparison vibration with frequency equal to 34 Hz plus or minus a value determined by the subject’s performance in the titration task. The presentation order of the base and comparison stimuli was randomly varied from trial-to-trial.

Two intermixed staircases were used, one for easy and one for hard levels. For both staircases, the difference in frequency was initially set to 5 Hz each then progressively decreased or increased by 10% of the current frequency difference across trials. An increase (step-up) or a decrease (step-down) in frequency difference depended on whether the subject responded correctly or incorrectly. For both staircases, a step-up occurred for each incorrect response. For the easy staircase, a step-down occurred after six non-consecutive correct responses. That is, even amongst trials of incorrect responses, a tally was kept for each correct response made. Once the tally reached six, a step-down occurred and the tally was reset to zero. Likewise, for the hard staircase, a step-down occurred after two non-consecutive correct responses (using a tally reaching two). We aimed to have performance converge at ∼85% and ∼65% proportion correct, respectively [52]. Since, in order to limit fatigue and loss of concentration, a large number of trials could not be used to titrate subjects, we considered some variation around these target values of performance as acceptable. To limit the subject from experiencing a learning effect from consecutive easy or consecutive hard trials, the two staircases were intermixed and the selection of a trial from both staircases was random.

The easy staircase could only terminate after the subject had performed 80 trials, whereas the hard staircase could only terminate after the subject had performed 40 trials. Each staircase ended when a sliding window of 20 trials reached the proportion correct targets of 85% and 65%. At this point, the average frequency difference for each unique step-up and step-down points within the window was chosen as the subjects’ titrated easy and hard frequency difference value to be used in the main task. As one staircase would terminate before the other, it would continue to step-up and down to the subjects’ responses until the second staircase terminated. A medium value that fell in between was determined with the geometric mean using the easy and hard frequency differences for each subject. This tallied with pilot data using the preceding up-down transform rule approach averaged to a target accuracy of 75%.

Following the titration task, noise was added to the temporal structure of the vibration pairs for a subset of trials in the main task. These were constructed by adding zero mean independent Gaussian-distributed values to each cycle of the sine wave [8]. We added 8% noise, meaning that the standard deviation of the cycle length within the vibration equalled 0.08 of the base cycle length. For example, a 40 Hz vibration was comprised of cycles with mean length 25 msec and standard deviation of 2 msec.

#### Main task

The main task, completed after the titration procedure, was performed in four separate sessions. For each trial, one of the vibrations was the base 32, 34 or 36 Hz. The value of the comparison frequency was determined by the subjects’ titrated easy, medium and hard frequency difference values and were either added to or subtracted from the base frequency. The order of the base and comparison frequency was counterbalanced. The selection of base frequency, the value of the comparison frequency, and the order of vibrations were pseudorandomly presented to the subject for each session. “Edge” vibrations with a base frequency of 30 Hz or 38 Hz and a comparison frequency determined by the subject’s easy frequency difference value, were also included in each session. Edge vibrations served to create comparison vibrations that were perceived as distinctly slow and distinctly fast by the subject. The objective of including edge vibrations at the edges of the stimulus-set was to ensure that the subjects compared the two vibrations for all other trials, rather than being able to make a categorical judgement about the frequency of the second vibration independently of the first [11].

For two sessions, the subjects had to answer the question, “Is the 2nd vibration faster?” (fast-slow context). For the other two sessions, the subject had to answer the question, “Are the vibrations different?” (same-diff context). For fast-slow sessions, subjects were told that there was always a faster vibration. For these two sessions, there were five trial-types; easy, medium, hard, easy noisy, medium noisy. For same-diff sessions, subjects were told that half of the presented vibration pairs were the same, and the other half were different. For these two sessions, there were six trial-types; easy different, medium different, easy noisy different, easy same, medium same, easy noisy same. If a subject’s frequency difference for easy trials was 10 Hz, and we consider a base frequency of 34 Hz, four possible easy same vibration pairs would have been produced in an equal number of trials: 34–34, 34–34, 24–24 and 44–44 (that is, two same trials had the base frequency repeated, and two where each comparison frequency was repeated). Likewise, with a medium frequency difference of 6 Hz, the pairs for medium same would be as follows: 34–34, 34–34, 28–28 and 40–40. Thus the easy same and medium same trials have unique vibration pairs when each respective comparison frequency was repeated, but both contain the same repeated base frequencies across half of their trials. Easy noise same trials contained two identical noise-embedded stimuli. The presentation order for each session was alternated and counterbalanced across subjects. There were 24 trials each across all four sessions for each of the 11 trial-types (see Table 1).

There were no significant differences between the subjects that used one button order (“Yes” is left, “No” is right) over the other (“Yes” is right, “No” is left) (d′: *F*_1, 58_ = 0.409, *p* = 5812; RT: *F*_1, 58_ = 1.334, *p* = 2529). Likewise, there were no significant differences between the subjects that were presented with one session arrangement (fast-slow, same-diff, fast-slow, same-diff) over the other (same-diff, fast-slow, same-diff, fast-slow) (d′: *F*_1, 58_ = 0.131, *p* = 7183; RT: *F*_1, 58_ = 0.008, *p* = 9270).

### Analysis 1

For the task difficulty, and the task difficulty and noise comparisons, d′ was used to assess accuracy. Details of how d′ was determined for both contexts are found further up in this Methods section under “Procedures”.

### Analysis 2

For the purposes of our dataset, the global mean of the stimulus-set was 34 Hz for the main task. The study used three base frequencies –32, 34 and 36 Hz. The comparison frequency was determined by the titrated frequency difference from each subject that was added to or subtracted from the base frequency. For every higher comparison frequency, there was a lower one. Hence on average the global mean was 34 Hz for each subject that performed the task.

Here we conducted two separate analyses to demonstrate that the time-order effect was having an influence on subjects’ decision making during the task. The first analysis compared preferred and nonpreferred time-order trials. For each subject, the average d′ of four of their preferred trials were compared to four of their nonpreferred trials. The second analysis compared base-first to base-second trials in order to exclude the possibility that base-order, which were not independent of preferred and nonpreferred trials, could explain the difference in performance of these two trial-types. This was achieved by confining an analysis of base position to nonpreferred trials only. The average of four base-first trials (Figure 2, panels A and G) were compared to the average of four base-second trials (Figure 2, panels B and H). If the position of the base frequency was the factor that influenced performance, then subjects would be expected to show greater accuracy for the base-first trials. Not all subjects’ data was used for the base-first and base-second comparison. A portion of the subjects had a titrated medium and/or hard frequency difference below 2 Hz, which creates a condition where an equal number of magnitude-matched nonpreferred trials of base-first and base-second was not available for comparison (see Figure S1 for details). By removing those participants that did not share the distribution of preferred and nonpreferred trials as required for a base-first and base-second comparison across all three levels of task difficulty (easy, medium, hard), this left 44 participants. There was a significant effect of task difficulty (*F*_2,86_ = 23.444, *p*<0001) but no significant difference for the base frequency position (*F*_1,43_ = 0.988, *p* = 3258). Thus, the position of the base frequency does not lead to any significant difference in accuracy when the influence of the time-order effect is removed (by looking at nonpreferred trials only).

In order to compare the influence of each factor of interest, we used partial eta squared (η^2^) as follows:
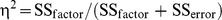
where the ratio of variance accounted for by the factor of interest was determined by the sum of squares of the factor of interest (SS_factor_) and the associated error variance sum of squares (SS_error_).

### Analysis 3

Along with factors task difficulty and noise, the time-order was included as an additional factor of interest. Only the behavioural response of the four preferred and four nonpreferred trials from each subject were compared. For the task difficulty and context contrast, the same trials of the same-diff context were excluded from analysis. This was done to directly compare the identical stimulus-set used for the fast-slow trials and the different trials of the same-diff context. That is, the subjects were exposed to the same stimuli, yet responded to a different contextual instruction, framing their decisions in a different manner across sessions. Since the same trials of the same-diff context were removed, d′ could not be determined. Same trials could be classified as false alarms (when subjects thought trials were different) for the same-diff context, and their removal from this analysis precluded the use of d′ which requires both hit and false alarm rates to be known. Instead, proportion correct was used to assess accuracy.

### Analysis 4

For the task difficulty (easy, medium, hard) and distance (closer, further) analysis, data from 44 out of the total 60 subjects were used as the remaining 16 subjects did to permit analysis (see for details). The same 44 subjects were used for the subsequent task difficulty (easy, medium), noise (regular, noisy) and distance (closer, further) analysis.

## Supporting Information

Figure S1 **Frequency differences that give rise to different proportions of preferred and nonpreferred time-order trials.** Each green and red arrow is a vibration pair of a single trial. The preferred (green arrows) and nonpreferred (red arrows) trials are classified based on the magnitude and relative orientations of Stim1, Stim2, the global mean (dashed line) and the resulting drift direction of Stim1 (blue arrows). Each subject in the study has three frequency difference values between pairs of vibrations corresponding to the task difficulty levels easy, medium and hard. If any of these values were greater or less-than 2 Hz, the proportion of preferred to nonpreferred trials differed. The top figure shows an example of trials that result with a 3 Hz difference between vibration pairs. In this arrangement, there are six nonpreferred trials and two preferred trials. Participants with this proportion of time-order trials permit all of the analyses as outlined in the study since each analysis (i.e. preferred verses nonpreferred, base-first verses base-second, closer verses further) has a sufficient number of trials for comparison. The lower figure shows an example of trials that result with a 1 Hz difference between vibration pairs. In this arrangement, there are four nonpreferred trials and four preferred trials. Participants with this proportion of time-order trials must be excluded from some of the study analyses. In Analysis 2 where base-first verses base-second was compared to demonstrate that the difference in performance between the time-order trials was not due to base position, 16 of the participants were excluded from the analysis, leaving 44 in total. The excluded participants had hard and or medium frequency difference values below 2 Hz, which reclassified the required nonpreferred trials for the analysis (these were of the type shown in Figure 2, panels B and H). Likewise, the 44 subjects were included for the distance comparisons as outlined in Analysis 4.
(TIF)
