# Supplementary figures and images for: Prior and Present Evidence: How Prior Experience Interacts with Present Information in a Perceptual Decision Making Task
Source: PLoS One. 2012 May 29;7(5):e37580. doi: 10.1371/journal.pone.0037580 (PMC3362626; doi:10.1371/journal.pone.0037580)

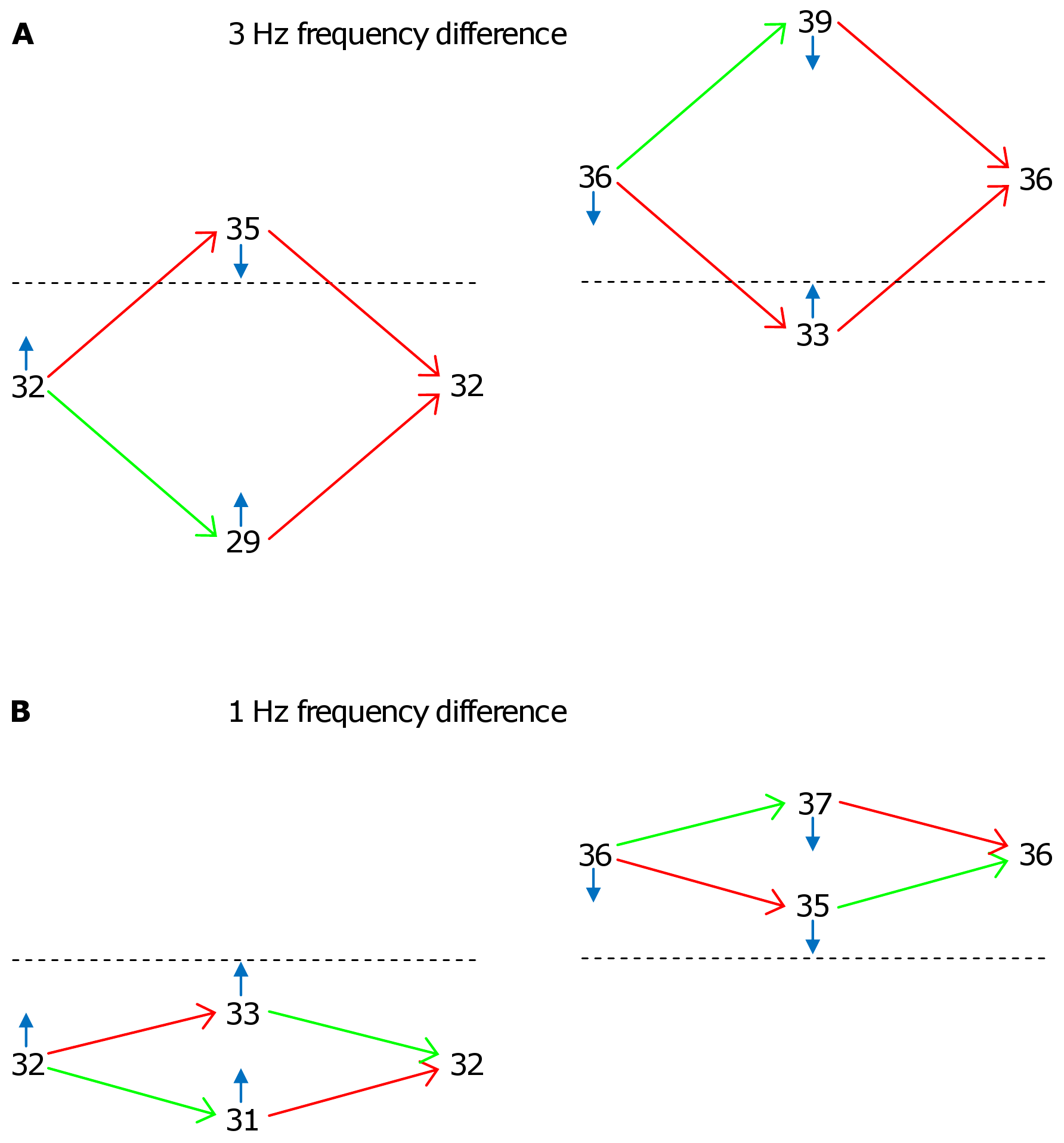

Supplement: Figure S1 — Frequency differences that give rise to different proportions of preferred and nonpreferred time-order trials. Each green and red arrow is a vibration pair of a single trial. The preferred (green arrows) and nonpreferred (red arrows) trials are classified based on the magnitude and relative orientations of Stim1, Stim2, the global mean (dashed line) and the resulting drift direction of Stim1 (blue arrows). Each subject in the study has three frequency difference values between pairs of vibrations corresponding to the task difficulty levels easy, medium and hard. If any of these values were greater or less-than 2 Hz, the proportion of preferred to nonpreferred trials differed. The top figure shows an example of trials that result with a 3 Hz difference between vibration pairs. In this arrangement, there are six nonpreferred trials and two preferred trials. Participants with this proportion of time-order trials permit all of the analyses as outlined in the study since each analysis (i.e. preferred verses nonpreferred, base-first verses base-second, closer verses further) has a sufficient number of trials for comparison. The lower figure shows an example of trials that result with a 1 Hz difference between vibration pairs. In this arrangement, there are four nonpreferred trials and four preferred trials. Participants with this proportion of time-order trials must be excluded from some of the study analyses. In Analysis 2 where base-first verses base-second was compared to demonstrate that the difference in performance between the time-order trials was not due to base position, 16 of the participants were excluded from the analysis, leaving 44 in total. The excluded participants had hard and or medium frequency difference values below 2 Hz, which reclassified the required nonpreferred trials for the analysis (these were of the type shown in Figure 2, panels B and H). Likewise, the 44 subjects were included for the distance comparisons as outlined in Analysis 4. (TIF) [file pone.0037580.s001.tif]
